# Lanthanum Affects Bell Pepper Seedling Quality Depending on the Genotype and Time of Exposure by Differentially Modifying Plant Height, Stem Diameter and Concentrations of Chlorophylls, Sugars, Amino Acids, and Proteins

**DOI:** 10.3389/fpls.2017.00308

**Published:** 2017-03-10

**Authors:** Atonaltzin García-Jiménez, Fernando C. Gómez-Merino, Olga Tejeda-Sartorius, Libia I. Trejo-Téllez

**Affiliations:** ^1^Plant Physiology, Colegio de Postgraduados Campus MontecilloMontecillo, Mexico; ^2^Biotechnology, Colegio de Postgraduados Campus CórdobaVeracruz, Mexico; ^3^Laboratory of Plant Nutrition, Soil Science, Colegio de Postgraduados Campus MontecilloMontecillo, Mexico

**Keywords:** Solanaceae, *Capsicum annuum*, rare earth elements, lanthanide, beneficial elements, biomolecules, seedling quality, hormesis

## Abstract

Lanthanum (La) is considered a beneficial element, capable of inducing hormesis. Hormesis is a dose-response relationship phenomenon characterized by low-dose stimulation and high-dose inhibition. Herein we tested the effect of 0 and 10 μM La on growth and biomolecule concentrations of seedlings of four sweet bell pepper (*Capsicum annuum* L.) varieties, namely Sven, Sympathy, Yolo Wonder, and Zidenka. Seedling evaluations were performed 15 and 30 days after treatment applications (dat) under hydroponic greenhouse conditions. Seedling height was significantly increased by La, growing 20% taller in Yolo Wonder plants, in comparison to the control. Similarly, La significantly enhanced shoot diameter, with increases of 9 and 9.8% in measurements performed 15 and 30 dat, respectively, as compared to the control. Likewise, La-treated seedlings had a higher number of flower buds than the control. An increase in the number of leaves because of La application was observed in Yolo Wonder seedlings, both 15 and 30 dat, while leaf area was augmented in this variety only 30 dat. Nevertheless, La did not affect dry biomass accumulation. La effects on biomolecule concentration were differential over time. In all varieties, La stimulated the biosynthesis of chlorophyll *a, b* and total 15 dat, though 30 dat only the varieties Sympathy and Yolo Wonder showed enhanced concentrations of these molecules because of La. Total soluble sugars increased in La-treated seedlings 30 dat. Interestingly, while most varieties exposed to La showed a reduction in amino acid concentration 15 dat, the opposite trend was observed 30 dat. Importantly, in all varieties evaluated, La stimulated soluble protein concentration 30 dat. It is important to note that while chlorophyll concentrations increased in all varieties exposed to La, both 15 and 30 dat, those of soluble sugars and proteins consistently increased only 30 dat, but not 15 dat. Our results confirm that La may improve seedling quality by enhancing some growth parameters and biomolecule concentrations, depending on the genotype, and time of exposure.

## Introduction

Lanthanum (La) is a metallic element belonging to the rare earth element (REE) group. It is commonly found in combination with cerium (Ce) and other elements of this group. It is a malleable, ductile and soft metal that is easily oxidized when exposed to air. It can be isolated from minerals such as monazite [(Ce, La, Th, Nd, Y)PO_4_] and bastnaesite [(Ce, La, Y)CO_3_F], in amounts ranging between 25 and 38% of the total lanthanide found (28 mg kg^−1^ of soil; Tyler, 2004).

In addition to its applications as a catalyst and in medicine, La has been employed in agriculture, where it has shown positive effects on plant physiology and improved some yield indicators in crops when applied at low concentrations (Hu et al., 2004). Nevertheless, this metal triggers hormesis, a dose-response phenomenon characterized by low-dose stimulation, high-dose inhibition. Thus, high La concentrations applied to plants may induce negative responses, instead of beneficial effects. In wheat (*Triticum aestivum*), the application of 0.5–25 mg L^−1^ La inhibited primary root elongation, reduced root, and shoot biomass accumulation, and disrupted the nutrient balance, especially that of Ca, Mg, K, Cu, and Zn (Hu et al., 2002). In maize (*Zea mays* cv. “Hycorn 82”) and mungbean (*Vigna radiata* cv. “Berken”), La decreased growth, root function, and nutritional status at concentrations >0.2 mM in solution (Diatloff et al., 2008). Moreover, in root tips of faba bean (*Vicia faba*) seedlings, La applied at concentrations higher than 0.5 mg L^−1^ induced nutrient imbalance of Ca, Fe and K, and DNA-protein crosslink, leading to hormetic response of cell cycle progression (Wang et al., 2012a). In horseradish (*Armoracia rusticana*), dual effects of La on cell growth were observed: low concentration (i.e., 30–35 μM LaCl_3_) promoted cell expansion, whereas high concentration (i.e., ≥ 80 μM LaCl_3_) inhibited cell expansion or even caused cell damage (Wang et al., 2014). In soybean (*Glycine max*), the application of 5–10 μM La in the nutrient solution improved plant performance, whereas 20–160 μM La drastically reduced photosynthetic rate and biomass accumulation (de Oliveira et al., 2015).

The differences in responses are attributed to the dose and application method, as well as the physical and chemical properties of the growth medium, the pH playing a key role. These responses are also related to the interaction with nutrients, type of crop and its developmental stage (Von Tucher and Schmidhalter, 2005; Zhang et al., 2013; de Oliveira et al., 2015; Turra et al., 2015).

Studies with La at seedling stage have focused on its protective effect against stress factors. For instance, La may mitigate the negative effects of heavy metals present in the growth medium. In fact, La may reduce the stress caused by Cd in common bean (*Phaseolus vulgaris*) and maize by improving the photosynthetic capacity, reducing membrane permeability and malondialdehyde (MDA, an indicator of oxidative stress in cells and tissue) content, and maintaining the activities of the antioxidant enzymes catalase (CAT) and peroxidase (POD) of these two crops (Huang and Zhou, 2006). Moreover, La influences the accumulation of trace elements such as Se, Co, Rb, V, Tc, and Ga in chloroplasts of cucumber (*Cucumis sativus*) seedling leaves by regulating ion transport mechanisms, while chloroplast contents peaks at 0.02 mM La^3+^, thus affecting photosynthesis (Zeng et al., 2000; Shi et al., 2006).

Lanthanum is first anchored on the plasma membrane in the form of nanoscale particles (Wang et al., 2014). Importantly, vitronectin-like protein (VN) is an anchoring site and binding target for La found on the plasma membrane of *Arabidopsis* leaf cells, and VN may act as a defense mechanism to resist the entry of La into plant cells (Yang et al., 2016). Once anchored on the plasma membrane, La may enter the cell by endocytosis, and a portion of it is released into the cytoplasm, self-assembled, and formed into nanoscale clusters. The endocytosis gradually ceases with the migration of La from leaf cells to leaf stalk, root, and soil (Wang et al., 2014). Furthermore, La affects the expression of calmodulin (CaM) in endocytosis. Low concentration of La could interact with extracellular CaM by electrostatic attraction and is then bound to two Ca-binding sites of CaM, making the molecular structure more compact and orderly, whereas a high concentration of La could be coordinated with cytoplasmic CaM or bound to other Ca-binding sites, making the molecular structure more loose and disorderly (Wang et al., 2016).

In horticultural production systems, seedling quality is a key factor determining an efficient crop performance. In turn, seedling quality is affected by a combination of characteristics of both a physical (i.e., height, diameter, health, root size, and shape, among others) and biochemical nature (i.e., soluble sugars, amino acids, and polyamines, including spermidine, spermine, and putrescine, among others; Ritchie, 1984; Lu et al., 2002; Vidigal et al., 2011). High quality seedlings have a higher survival rate and faster growth in the field than poor quality ones. In turn, fast growth allows crop plants to outcompete weeds and reduces the initial labor costs of establishment, enabling farmers to harvest products sooner and thus increase their return on investment (Resh, 2012). In particular, the initial growth phase in sweet bell pepper seedlings is the most critical stage for the commercial production of this crop species (De Grazia et al., 2004) and beneficial elements such as La may boost its performance. Herein we aimed to evaluate the effect of adding 10 μM LaCl_3_ in the nutrient solution on quality components of seedlings obtained from four sweet bell pepper varieties.

## Materials and methods

### Treatment design and experimental design

We germinated seeds of four commercial varieties of sweet bell pepper (*Capsicum annuum* L.), namely Sven, Sympathy, Yolo Wonder, and Zidenka. Some of the main agronomic descriptors of these genotypes are detailed in Supplementary Material 1. Germination was carried out in a mixture of peat-moss and perlite (80/20, v/v).

Fifteen days after emergence, seedlings were transferred to 500 mL pots containing perlite as substrate, and irrigated with Steiner's nutrient solution at 15% of its original strength (Steiner, 1984) for 15 days. Thirty days after emergence, the application of La contained in the nutrient solution was initiated. La was applied at concentrations of 0 and 10 μM and was supplied from LaCl_3_. Nutrient solutions were prepared using distilled water and analytical grade reagents (Sigma Adlrich; Darmstadt, Germany) at pH 5.5 and an electrical conductivity of 0.3 dS m^−1^. Under low pH value, the mobility and subsequent availability of La as cation increases (Von Tucher and Schmidhalter, 2005).

The macronutrient composition of Steiner's nutrient solution (at 15% of its original strength) was (in cmol_(+)_ m^−3^): 1.80 ${\text{NO}}_{3}^{-}$, 0.15 H_2_${\text{PO}}_{4}^{-}$, 1.05 ${\text{SO}}_{4}^{-2}$, 1.05 K^+^, 1.35 Ca^+2^, and 0.60 Mg^+2^. We used the diluted solution (15%) and not the full-strength version (100%) in order to avoid interactions of La with other cations such as Ca and Mg, in accordance with recommendations made by Kinraide (1998). The nutrient solution was supplemented with micronutrients at the following concentrations (in μM): 29.12 Mn, 1.73 Cu, 79.56 B, 0.35 Zn, and 0.50 Mo. Iron was supplied as Fe-EDTA at a concentration of 89.53 μM from a stock solution prepared according to Steiner and van Winden (1970).

Each experimental unit consisted of five seedlings in a 500 mL pot. All units were watered daily using 300 mL of diluted Steiner's nutrient solution. The total amount of the nutrient solution (300 mL) was fractionated in three watering events every 8 h, of 100 mL each.

The experiment was performed under hydroponic greenhouse conditions, testing two treatments on each variety of bell sweet peppers: 0 (control) and 10 μM La in the nutrient solution. The experiment had a completely randomized design. Each experimental unit consisted of five seedlings planted in a 500 mL pot with perlite as substrate. During the experiment, seedlings were grown under a day-length of 12 h, at 28/18°C day/night temperature, 550 μmol m^−2^ s^−1^ light intensity, and 70% relative humidity.

### Variables evaluated

Fifteen and 30 days after the application of treatments (dat), the following variables were evaluated: plant height, stem diameter, number of leaves, number of flower buds, leaf area and leaf, root and total biomass dry weight.

A leaf area integrator (LICOR, LI-300, Lincoln, NE, USA) was used for measuring leaf area, which was reported in cm^2^, while the dry weights were determined after drying the plant material for 48 h at 70°C in a forced air oven (Riossa, HCF-125D; Guadalajara, Jalisco, Mexico), using an analytical balance (Adventurer Pro AV213C, Ohaus; Parsippany, NJ, USA).

In all cases, the readings of the biochemical variables were made using a Thermo Scientific Genesys 10 spectrophotometer (Madison, WI, USA).

In fresh leaves the concentrations of chlorophylls *a, b* and total were determined as described by Harborne (1973) by spectrophotometry at a wavelength of 663 and 645 nm.

The concentration of total soluble sugars was determined in fresh leaves by the anthrone method described by Southgate (1976), and read in the spectrophotometer at 600 nm, using a glucose standard curve as reference.

Determination of free amino acids in fresh leaves was achieved by a triple ethanolic extraction, as described by Geiger et al. (1998). The extracts were read in the spectrophotometer at a 570 nm wavelength, using the ninhydrin method (Moore and Stein, 1954). Leucine was used for the preparation of the calibration curve.

Protein concentrations in leaves were determined according to the procedure described by Höfner et al. (1989). Reading of the samples was performed in the spectrophotometer with 640 nm absorbance. Bovine serum albumin was used to prepare the standard curve.

### Statistical analysis

The variables were analyzed by sampling date (i.e., either 15 or 30 dat) in each variety of bell sweet peppers, according to the treatments established. Analysis of variance and Tukey's range test were performed with a 95% confidence level using Statistical Analysis System (SAS, 2011) software.

## Results

**Figure 1:**
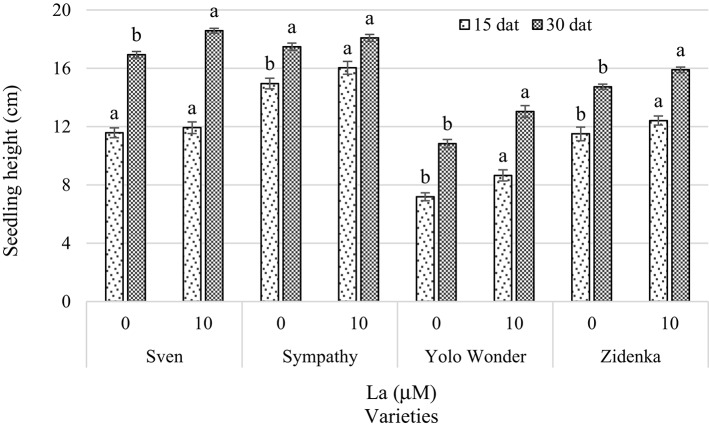
**Seedling height of four sweet bell pepper varieties treated with lanthanum (0 and 10 μM LaCl_**3**_) in the nutrient solution**. Measurements were carried out 15 and 30 days after treatment (dat). Means ± *SD* in each time of measurement and in each variety with different letters indicate statistical differences (Tukey, *P* ≤ 0.05). *P* > *F*-values at 15 dat are 0.2640, 0.0035, <0.0001, and 0.0105; and at 30 dat are <0.0001, 0.0580, 0.0003, and 0.0002, for Sven, Sympathy, Yolo Wonder, and Zidenka varieties, respectively.

In preliminary experiments, we tested the effect of a number of La concentrations on plant growth, development, and nutrient concentration (Ramírez-Martínez et al., 2009, 2012). The stimulant effect of La on plant growth was observed when this element was applied at concentrations between 10 and 20 μM La. Furthermore, we analyzed the literature on La dosage triggering beneficial effects in some important horticultural crops (Supplementary Material 2). In tomato (*Solanum lycopersicum*), the application of 40.78 μM La in the nutrient solution increased yield and reduced Cd accumulation in fruits (Xie et al., 2014). Similarly, Hu et al. (2014) demonstrated that the application of 4.08 μM La could efficiently protect pepper seedlings from salinity (0.75 g L^−1^ NaCl) damage by decreasing its death rate, increasing POD activity, and reducing DNA methylation. In cucumber (*Cucumis sativus*), the application of 10–20 μM La stimulated the antioxidant system and enhanced plant growth. Just recently, Trejo-Téllez et al. (2016) published a review on the effects of beneficial elements in crop plants. Based on those reports and our experience, we decided to perform further analysis by comparing the effect of applying 0 (control) and 10 μM La on plant growth and concentrations of chlorophylls, soluble sugars, amino acids and soluble proteins in sweet pepper seedlings. Commercial sweet pepper varieties Sven, Sympathy, Yolo Wonder and Zidenka were grown in Steiner's nutrient solution at 15% of its original strength under greenhouse conditions. Treatments without and with La (0 and 10 μM LaCl_3_, respectively) were applied to 30-day-old pepper seedlings for either 15 or 30 days. We found that plant height was stimulated by La, though significant effects were only evident in Sympathy, Yolo Wonder, and Zidenka 15 dat, and in Sven, Yolo Wonder, and Zidenka 30 dat (Figure 1).

**Figure 2:**
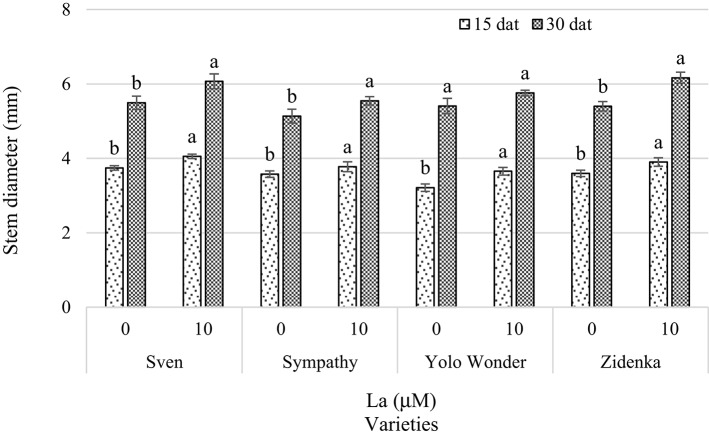
**Stem diameter of seedlings of four sweet bell pepper varieties treated with lanthanum (0 and 10 μM LaCl_**3**_) in the nutrient solution**. Measurements were carried out 15 and 30 days after treatment (dat). Means ± *SD* in each time of measurement and in each variety with different letters indicate statistical differences (Tukey, *P* ≤ 0.05). *P* > *F*-values at 15 dat are <0.0001, 0.0389, <0.0001, and 0.0012; and at 30 dat are 0.0251, 0.0434, 0.0775, and 0.0007, for Sven, Sympathy, Yolo Wonder, and Zidenka varieties, respectively.

Stem diameter evaluated 15 dat was statistically different in all varieties, with La increasing this variable by 9% on average, in comparison to the control. In measurements performed 30 dat, in Sven, Sympathy, Zidenka, but not in Yolo Wonder, La increased stem diameter by 9.8%, as compared to control seedlings (Figure 2).

Interestingly, flower buds appeared on the seedlings 30 dat. The number of flower buds was significantly increased in the presence of La, with the exception of Sympathy seedlings. In general, a positive effect of La on flower bud induction was observed. In all varieties except Yolo Wonder, La did not affect the number of leaves per plant (Table 1).

**Table 1:** **Number of flower buds and leaves per plant of four sweet bell pepper varieties treated with La (0 and 10 μM LaCl_**3**_) in the nutrient solution**.

**Variety**	**La (μM)**	**Number of flower buds per plant, 30 dat**	**Number of leaves per plant**	
			**15 dat**	**30 dat**
Sven	0	3.00 ± 0.41b	11.50 ± 0.87a	18.75 ± 1.18a
Sven	10	5.00 ± 0.41a	12.50 ± 0.65a	19.75 ± 1.89a
*Pr* >F		0.0134	0.3903	0.6691
Sympathy	0	3.00 ± 0.41a	13.50 ± 1.04a	18.25 ± 0.85a
Sympathy	10	4.25 ± 0.48a	12.50 ± 0.65a	18.75 ± 0.75a
*Pr* >F		0.0941	0.4454	0.6754
Yolo Wonder	0	0.25 ± 0.25b	9.75 ± 0.48b	15.00 ± 0.91b
Yolo Wonder	10	1.75 ± 0.48a	11.50 ± 0.29a	19.00 ± 1.29a
*Pr* >F		0.0321	0.0203	0.0447
Zidenka	0	1.25 ± 0.25b	9.25 ± 0.48a	14.00 ± 0.71a
Zidenka	10	2.75 ± 0.48a	9.50 ± 0.65a	16.25 ± 1.11a
*Pr* >F		0.0321	0.7663	0.1379

*Means ± SD in each time of measurement (i.e., 15 and 30 dat) and variety with different letters indicate statistical differences (Tukey, P ≤ 0.05). dat, days after treatment application*.

**Figure 3:**
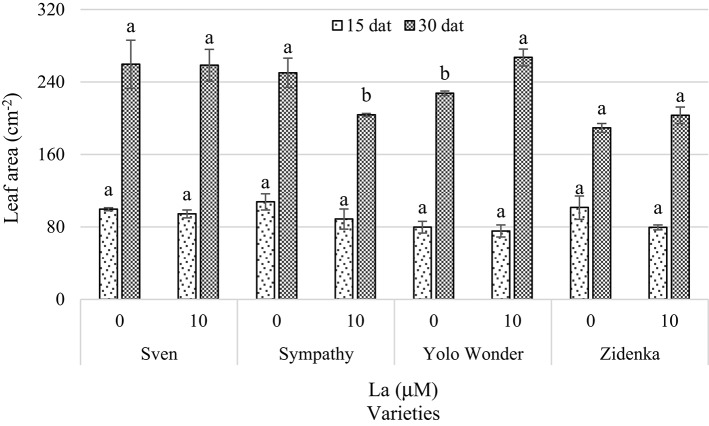
**Leaf area of seedlings of four sweet bell pepper varieties treated with lanthanum (0 and 10 μM LaCl_**3**_) in the nutrient solution**. Measurements were carried out 15 and 30 days after treatment (dat). Means ± *SD* in each time of measurement and in each variety with different letters indicate statistical differences (Tukey, *P* ≤ 0.05). *P* > *F*-values at 15 dat are 0.3135, 0.2306, 0.1483, and 0.6615; and at 30 dat are 0.9694, 0.0280, 0.0067, and 0.2275, for Sven, Sympathy, Yolo Wonder, and Zidenka varieties, respectively.

Concerning leaf area, La did not affect this component 15 dat. However, La did reduce this variable in the variety Sympathy (18.6% in comparison to the control), whereas the variety Yolo Wonder showed an increased leaf area (17.3% as compared to the control) 30 dat in response to La (Figure 3).

**Figure 4:**
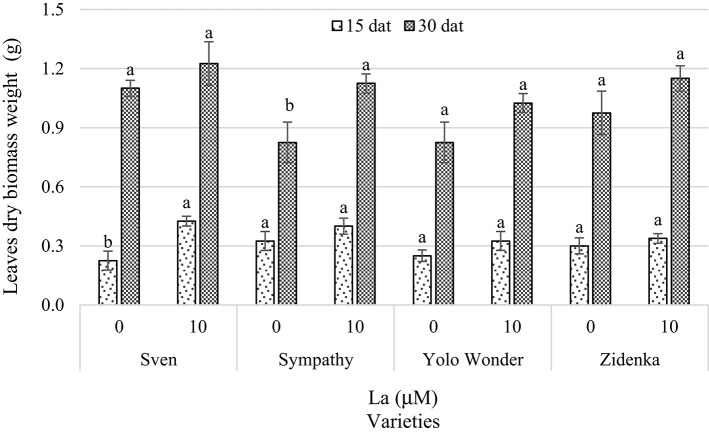
**Leaf dry biomass weight of seedlings of four sweet bell pepper varieties treated with lanthanum (0 and 10 μM LaCl_**3**_) in the nutrient solution**. Measurements were carried out 15 and 30 days after treatment (dat). Means ± *SD* in each time of measurement and in each variety with different letters indicate statistical differences (Tukey, *P* ≤ 0.05). *P* > *F*-values at 15 dat are 0.0100, 0.2782, 0.2283, and 0.4583; and at 30 dat are 0.3308, 0.0386, 0.1289, and 0.2215, for Sven, Sympathy, Yolo Wonder, and Zidenka varieties, respectively.

Leaf dry biomass was enhanced by La application in most varieties evaluated, though significant differences between control and La-treated plants were evident only in the variety Sven 15 dat and Sympathy 30 dat. Although no significant differences were found 30 dat in three of the four varieties evaluated, it is worth noting that La increased leaf dry biomass weight by 11, 36, 24, and 18% in the varieties Sven, Sympathy, Yolo Wonder, and Zidenka, respectively, compared to their respective controls, with Sympathy being the only one showing statistical differences at this time of measurement (Figure 4).

**Figure 5:**
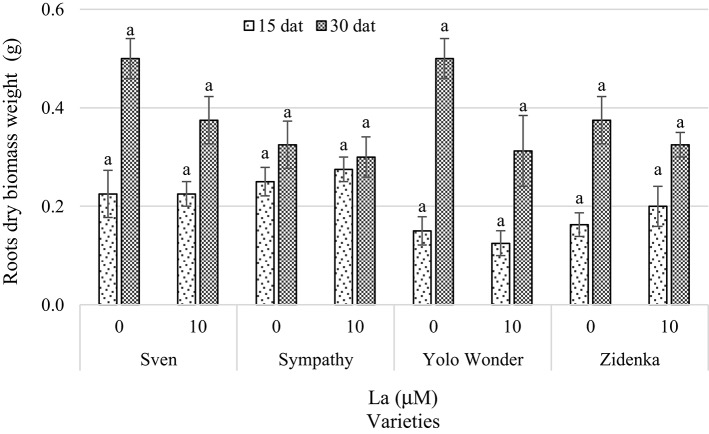
**Root dry biomass weight of seedlings of four sweet bell pepper varieties treated with lanthanum in the nutrient solution**. Measurements were carried out 15 and 30 days after treatment (dat). Means ± *SD* in each time of measurement and in each variety with different letters indicate statistical differences (Tukey, *P* ≤ 0.05). *P* > *F*-values at 15 dat are 1.0000, 0.5370, 0.5370, and 0.4583; and at 30 dat are 0.0941, 0.7049, 0.0637, and 0.3903, for Sven, Sympathy, Yolo Wonder, and Zidenka varieties, respectively.

Interestingly, La did not affect root dry biomass weight in any of the varieties evaluated during both sampling times (i.e., 15 and 30 dat) as compared to the control (Figure 5).

**Figure 6:**
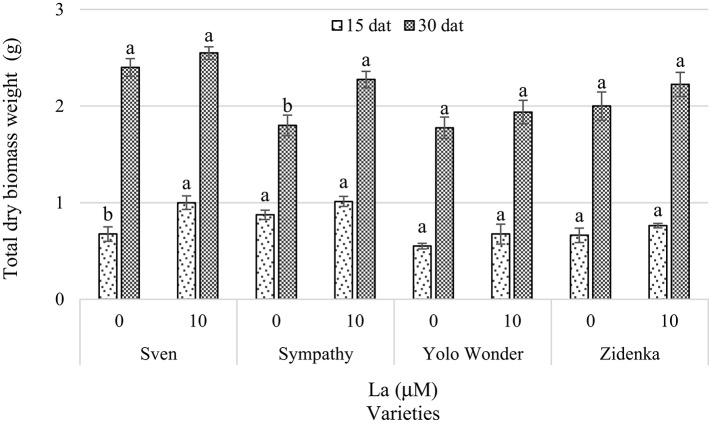
**Total dry biomass weight of seedlings of four sweet bell pepper varieties treated with lanthanum (0 and 10 μM LaCl_**3**_) in the nutrient solution**. Measurements were carried out 15 and 30 days after treatment (dat). Means ± *SD* in each time of measurement and in each variety with different letters indicate statistical differences (Tukey, *P* ≤ 0.05). *P* > *F*-values at 15 dat are 0.0197, 0.0984, 0.2872, and 0.2493; and at 30 dat are 0.2283, 0.0136, 0.3611, and 0.2282, for Sven, Sympathy, Yolo Wonder, and Zidenka varieties, respectively.

Significant effects of La applications on total dry biomass weight were only observed in the Sven variety 15 dat, with an increase of 48% as compared to the control. In the other varieties measured 15 dat, adding La slightly increased total dry biomass weight compared to their respective controls, but these increases were not significant. Thirty days after treatment, we observed a significant increase (26.4%) in total dry biomass weight only in Sympathy, in comparison to the control, whereas the other three varieties did not show any effect of La regarding this variable (Figure 6).

Table 2 shows the concentration of chlorophylls measured 15 and 30 dat. Fifteen days after treatment, concentrations of chlorophylls *a, b* and total significantly increased in all four varieties exposed to La. Importantly, in Yolo Wonder seedlings, concentrations of chlorophylls *a, b* and total were 120, 132, and 119% higher, respectively, in comparison to the control. Conversely, 30 dat we observed different responses. On one hand, Sven and Zidenka seedlings were not affected by La applications. On the other hand, in Sympathy and Yolo Wonder seedlings, La reduced the concentrations of chlorophyll *a* by 39.3 and 19.6%, chlorophyll *b* by 53.5 and 13.1%, and total chlorophylls by 44.4 and 17.7%, respectively, in comparison to the control (Table 2).

**Table 2:** **Chlorophylls concentration in leaves of four sweet bell pepper varieties as affected by varieties treated with La (0 and 10 μM LaCl_**3**_) in the nutrient solution**.

**Variety**	**La (μM)**	**Chlorophyll *a* (mg g ^−1^ FBW)**	**Chlorophyll *b* (mg g ^−1^ FBW)**	**Chlorophyll total (mg g ^−1^ FBW)**
**15 dat**				
Sven	0	0.123 ± 0.004b	0.045 ± 0.001b	0.170 ± 0.004b
Sven	10	0.210 ± 0.006a	0.079 ± 0.002a	0.293 ± 0.006a
*Pr* >F		<0.0001	<0.0001	<0.0001
Sympathy	0	0.190 ± 0.009b	0.070 ± 0.001b	0.263 ± 0.009b
Sympathy	10	0.295 ± 0.011a	0.136 ± 0.009a	0.435 ± 0.008 a
*Pr* >F		0.0004	0.0003	<0.0001
Yolo Wonder	0	0.059 ± 0.002b	0.025 ± 0.001b	0.086 ± 0.002b
Yolo Wonder	10	0.130 ± 0.004a	0.058 ± 0.001a	0.189 ± 0.004a
*Pr* >F		<0.0001	<0.0001	<0.0001
Zidenka	0	0.063 ± 0.001b	0.023 ± 0.001b	0.086 ± 0.001b
Zidenka	10	0.129 ± 0.003a	0.053 ± 0.002a	0.184 ± 0.003a
*Pr* >F		<0.0001	<0.0001	<0.0001
**30 dat**				
Sven	0	0.263 ± 0.029a	0.154 ± 0.002a	0.420 ± 0.030a
Sven	10	0.242 ± 0.032a	0.164 ± 0.007a	0.409 ± 0.039a
*Pr* >F		0.6464	0.2416	0.8256
Sympathy	0	0.191 ± 0.018a	0.101 ± 0.002a	0.295 ± 0.016a
Sympathy	10	0.116 ± 0.009b	0.047 ± 0.001b	0.164 ± 0.009b
*Pr* >F		0.0085	<0.0001	0.0005
Yolo Wonder	0	0.225 ± 0.013a	0.099 ± 0.002a	0.327 ± 0.015a
Yolo Wonder	10	0.181 ± 0.011b	0.086 ± 0.002b	0.269 ± 0.012b
*Pr* >F		0.0413	0.0015	0.0210
Zidenka	0	0.239 ± 0.033a	0.160 ± 0.006a	0.402 ± 0.037a
Zidenka	10	0.285 ± 0.011a	0.145 ± 0.006a	0.433 ± 0.017a
*Pr* >F		0.2375	0.1115	0.4706

*Means ± SD in each time of measurement (i.e., 15 and 30 dat) and in each variety with different letters indicate statistical differences (Tukey, P ≤ 0.05). dat, days after treatment; FBW, Fresh Biomass Weight*.

**Figure 7:**
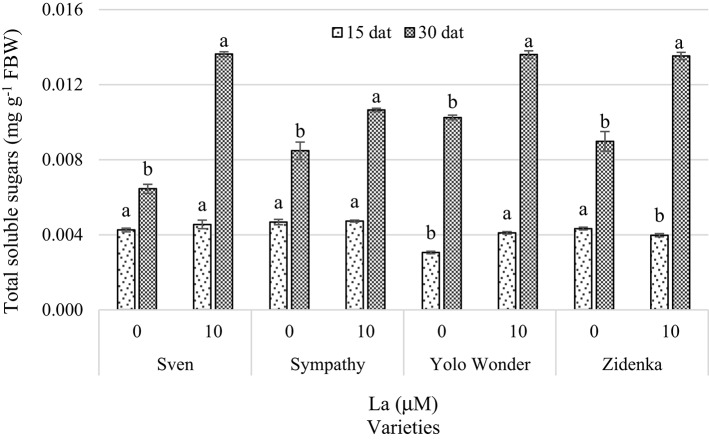
**Total soluble sugars in leaves of seedlings of four sweet bell pepper varieties treated with lanthanum (0 and 10 μM LaCl_**3**_) in the nutrient solution**. Measurements were carried out 15 and 30 days after treatment (dat). Means ± *SD* in each time of measurement and in each variety with different letters indicate statistical differences (Tukey, *P* ≤ 0.05). *P* > *F*-values at 15 dat are 0.2839, 0.7524, <0.0001, and 0.0217; and at 30 dat are <0.0001, 0.0037, <0.0001, and 0.0002, for Sven, Sympathy, Yolo Wonder, and Zidenka varieties, respectively. FBW, Fresh Biomass Weight.

In measurements performed 15 dat, La application significantly enhanced total soluble sugars in Yolo Wonder, whereas Zidenka displayed a significant decrease in response to La (Figure 7). In the varieties Sven and Sympathy, La had no significant effects on total soluble sugar concentrations 15 dat, though 7 and 1% increases were observed in both varieties, respectively, in comparison to the control. Interestingly, a positive response to La was observed in all four varieties evaluated 30 dat. In this evaluation, the Sven, Sympathy, Yolo Wonder, and Zidenka varieties showed increases of 111, 25, 32, and 50%, respectively, compared to the corresponding controls (Figure 7).

**Figure 8:**
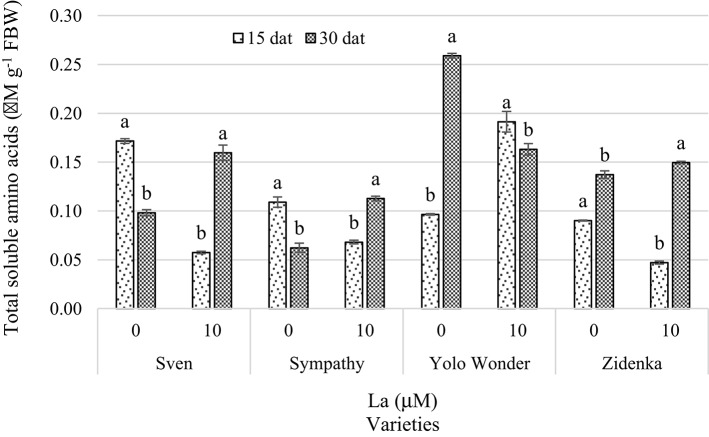
**Total soluble amino acids in leaves of seedlings of four sweet bell pepper varieties treated with lanthanum (0 and 10 μM LaCl_**3**_) in the nutrient solution**. Measurements were carried out 15 and 30 days after treatment (dat). Means ± *SD* in each time of measurement and in each variety with different letters indicate statistical differences (Tukey, *P* ≤ 0.05). *P* > *F*-values at 15 dat are <0.0001, 0.0003, 0.0001, and <0.0001; and at 30 dat are 0.0004, <0.0001, <0.0001, and 0.0226, for Sven, Sympathy, Yolo Wonder, and Zidenka varieties, respectively. FBW, Fresh Biomass Weight.

The effects of La on the concentration of total amino acids were differential over time. Fifteen days after treatment, La exerted a negative effect on the Sven, Sympathy, and Zidenka varieties. The average reduction in this variable was 53%, compared to the control. By contrast, 30 dat in Sven, Sympathy, and Zidenka seedlings the application of La increased this variable, especially in Sven and Sympathy, with an ~71.8% increase compared to the control (Figure 8).

**Figure 9:**
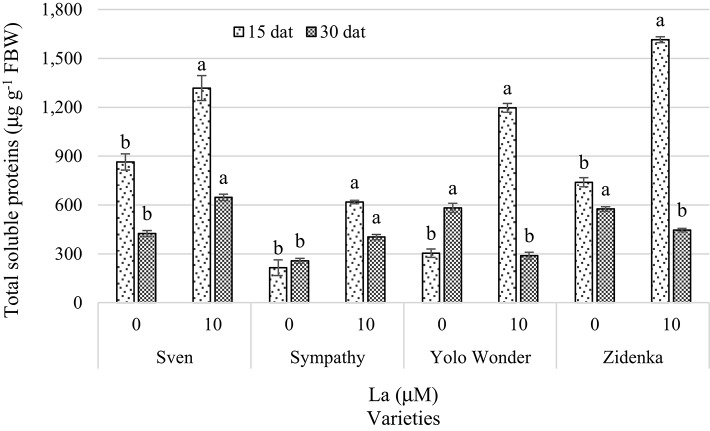
**Total soluble proteins in leaves of seedlings of four sweet bell pepper varieties treated with lanthanum (0 and 10 μM LaCl_**3**_) in the nutrient solution**. Measurements were carried out 15 and 30 days after treatment (dat). Means ± *SD* in each time of measurement and in each variety with different letters indicate statistical differences (Tukey, *P* ≤ 0.05). *P* > *F*-values at 15 dat are 0.0025, 0.0002, <0.0001, and <0.0001; and at 30 dat are 0.0002, 0.0006, 0.0002, and 0.0004, for Sven, Sympathy, Yolo Wonder, and Zidenka varieties, respectively. FBW, Fresh Biomass Weight.

Fifteen days after treatment, the concentration of soluble proteins increased significantly in all La-treated varieties compared to the control. This trend was still evident in measurements made 30 dat in the Sven and Sympathy varieties. On the other hand, at 30 dat the Yolo Wonder and Zidenka varieties showed a significant reduction in the concentration of soluble proteins due to the effect of La, with 50.3 and 22.4% lower protein concentrations, respectively, as compared to the control (Figure 9).

## Discussion

The group of REE consists of 17 elements, including lanthanum. These elements have been widely used in agriculture over the last 30 years and the largest REE producers are China, Russia, and Brazil (Pathan et al., 2013). These elements have not been characterized as essential to life, or as strongly toxic elements in the environment and their use at low concentrations can promote the growth and productivity of various crops (Tyler, 2004). Thus, La may potentially contribute to hormetic effects in various biological parameters in a dose-response manner (Wang et al., 2012a).

In this study we evaluated the effect of lanthanum on growth and biochemical variables in seedlings of four sweet bell pepper varieties, among which significant effects of La were found. The addition of 10 μM La to the nutrient solution positively affected plant height in three of the four varieties evaluated 15 and 30 dat (Figure 1). As well, stem diameter was significantly increased in three varieties exposed to La in the two samplings carried out 15 and 30 dat (Figure 2). The positive effect of La on plant growth has been reported in other species. For instance, He and Loh (2000) found that the addition of lanthanum nitrate to the growth medium triggered a striking increase in the length of primary roots of *Arabidopsis thaliana* during the vegetation stage. In rice (*Oryza sativa*), Zeng et al. (2006) found that supplying La^3+^ to a cambisol soil at 30–300 mg kg^−1^ increased plant height and the number of shoots during the vegetative stage. In *Vigna radiata, V. mungo*, and maize, the application of 5–50 μM La in the nutrient solution enhanced the percentage of germination and increased root length and plant height (Chaturvedi et al., 2014). In tulip (*Tulipa gesneriana*) cv. Ile de France, diameter and length of flowers significantly increased with the supply of 10 μM of La(NO_3_)_3_ (Ramírez-Martínez et al., 2009).

Lanthanum increased the number of flower buds in Sven, Yolo Wonder, and Zidenka varieties 30 dat (Table 1). Accordingly, He and Loh (2000) reported that applications of 0.5 μM La induced flowering in *Arabidopsis thaliana*. Subsequently, it was demonstrated that such induction was correlated with increased endogenous concentrations of cytokinins (i.e., isopentenyl adenosine; He and Loh, 2002), which are phytohormones regarded as important components of floral stimuli in plants. These results indicate that La may have the potential to be developed as non-hormonal flowering promoting agents in certain plant species.

Under our experimental conditions, La did not affect the number of leaves, except in the variety Yolo Wonder. In this variety, La increased the number of leaves by two and four, 15 and 30 dat, respectively, in comparison to the control (Table 1). Coincidently, only this variety showed a positive effect of La on leaf area 30 dat, since this component increased by 17.3% in La-treated plants in comparison to the control (Figure 3). Additionally, La did not affect leaf dry biomass weight in most varieties evaluated, with the exception of Sympathy 30 dat (Figure 4). Similarly, Xie et al. (2014) reported different growth responses to La in two varieties of tomato (i.e., 4641 and Yufen109). Therefore, it is assumed that La may exert different effects on plant growth depending on the genotype tested.

Although the presence of La in the nutrient solution reduced root biomass in all four pepper varieties evaluated 30 dat, this reduction was not significant (Figure 5). In wheat the addition of both La and Ce and their combination at concentrations between 0.5 and 25 mg L^−1^ in the nutrient solution inhibited the length of the primary root and reduced root and shoot dry biomass (Hu et al., 2002). Conversely, Liu and Hasenstein (2005) found greater root growth in corn treated with La^3+^ due to the stabilization of the cytoskeleton caused by this element when entering the cell. In rice cv. Shengdao 16 seedlings, Liu et al. (2013) reported positive effects of La when applied at 0.05, 0.1, and 0.5 mmol L^−1^, while by increasing these levels to 1.0 and 1.5 mmol L^−1^ La^3+^, growth inhibition was observed 13 days after treatment. Furthermore, Zeng et al. (2006) found that the application of 30 mg La^+3^ kg^−1^ of red soils (haplic acrisols) enhanced shoot biomass and yield, while the highest root weight was found with 150 mg La^3+^ kg^−1^ of soil. In paddy soils (cambisols), no differences were observed regarding these variables by applying 0–300 mg La^3+^ kg^−1^ of soil. However, in both types of soil a supply greater or equal to 600 mg kg^−1^ La decreased rice growth and yield. In Rangpur lime (*Citrus limonia* Osbeck), adding 50 mg of LaCl_3_ dissolved in 100 mL of water to each plant increased plant biomass and height, but higher concentrations inhibited growth (Turra et al., 2015). Xiong et al. (2006) found the longest root length in rapeseed (*Brassica napus*) plants when they were treated with 0.25 mg L^−1^ La^3+^, while the dry matter was greater with 1 and 5 mg L^−1^ La^3+^. In soybean, low La concentrations (i.e., 5–10 μM) stimulated the photosynthetic rate and total chlorophyll content and led to a higher incidence of binucleate cells, resulting in a slight increase in root and shoot biomass. At higher La levels (>20 μM), soybean growth was reduced, as a consequence of ultrastructural modifications in the cell wall, thylakoids and chloroplasts, and the appearance of c-metaphases (de Oliveira et al., 2015). Indeed, La affects root growth, at least partially, by modulating ROS levels in roots to induce cell death in primary root tips and subsequent auxin redistribution in roots, leading to remodeling of the root system architecture (Liu et al., 2016). Thus, La may exert different modes of action not only among genotypes but also among the phenological phases and organs evaluated.

Beneficial effects of La on plant growth and biomass accumulation, when applied at low concentrations, may be attributed to the signal cascades it triggers, including an improved performance of PSII, a higher velocity of electron transport during photosynthesis, a higher accumulation of mineral nutrients in the chloroplast, an enhanced activity of ATPases, and higher photosynthetic rates (Hong et al., 2002; Hu et al., 2016a). Conversely, higher doses of La may unbalance cell homeostasis, by causing damage in the external membrane of the chloroplast, decreasing the concentrations of mineral nutrients in this organelle, especially those of P, Mg, K, Ca, Mn, Fe, Ni, Cu, Zn, and Mo, and disrupting chlorophyll biosynthesis. Altogether, such negative effects of high La concentrations may lead to a drastic reduction in photosynthesis, which in turn causes lower growth and negative impacts on plant performance in general (Hu et al., 2016b). In the common freshwater microalga *Desmodesmus quadricauda* subjected to Ca deficit, the addition of La partly alleviated the adverse condition of the macronutrient shortage, probably by a partial substitution of Ca by La. However, with Mg deprivation (and at even lower concentrations), La enhanced the deleterious effect on cellular growth and photosynthetic competence. Hence, La can replace essential elements such as Ca and Mg, but its effects on microalgae depend on the stress and the nutritional state of the organism, raising the possibility of environmental impacts at even low concentrations (Goecke et al., 2015).

With respect to the biomolecule concentrations in the leaves of the sweet bell pepper varieties under study, changes were observed over time (Table 2; Figures 7–9).

Chlorophylls are the predominant pigment in the photosynthetic process. It has been found that the higher their concentration, the better the rate of CO_2_ sequestration and thus the higher the growth rate (Rathore and Jasrai, 2013). Under our experimental conditions, La significantly increased chlorophylls concentration in all four varieties evaluated 15 dat. On the contrary, 30 dat, the concentrations of such molecules was reduced or not affected by La (Table 2). According to Chaturvedi et al. (2014), the application of 5–50 μM La in *Vigna radiata, V. mungo*, and maize increased the concentrations of chlorophylls *a, b* and total. This response is explained by the fact that La may substitute Mg^2+^ during the formation of chlorophyll molecules (Hong et al., 2002; Goecke et al., 2015) and therefore more atoms for the formation of the chlorophyll molecule could be available in the growth medium. Furthermore, La may change the morphology of the external membrane of the chloroplast, which improves the structure of the organelle and leads to a higher concentration of functional nutrients such as N in the chloroplast (Hu et al., 2016b). Importantly, N is also a key component in the structure of the chlorophyll molecule. In our study, a slight reduction of chlorophyll *a* concentration was observed in the variety Sven 30 dat in response to La addition, with a concomitant reduction in the chlorophyll *a*/*b* ratio. Nevertheless, this reduction was not statistically significant (Table 2). A reduction in the chlorophyll *a*/*b* ratio associated with the treatment with high La concentrations in rice may be a response to stress, since the La concentrations ≥ 600 mg kg^−1^ dry soil increased membrane permeability, proline content and POD activity, all of which are indicators of physiological stress (Zeng et al., 2006). In the green alga *Desmodesmus quadricauda*, the application of 10 μM LaCl_3_ enhanced growth by as much as 36% compared to the control, the photosynthetic rate increased by up to 300%, and the total chlorophyll concentration also rose, especially because of a significant increase in chlorophyll *b* concentration (Řezanka et al., 2016). Nevertheless, the ratio of chlorophyll *a*/*b* in the fern *Dicranopteris dichotoma* decreased with increasing content of La in the soil (Wang et al., 2005), which suggests a negative impact of La on plant metabolism and, therefore, a stress signal triggered by this lanthanide.

Plants are integrated systems of photosynthetic carbon sources and non-photosynthetic carbon-consuming sinks, so the increase in the concentration of sugars in leaves represents the source that supplies the requirements for the shoot and roots, supporting vital processes such as growth, development, and reproduction (McCormick et al., 2009). Leaves are the major photosynthetic sources, with chlorophylls playing a pivotal role during energy absorption from light. Such energy is ultimately used to reduce CO_2_ into sugars (i.e., sucrose) during photosynthesis. Sucrose is the main photo-assimilate translocated from source to sinks, via phloem tissues. Our results do not show a direct correlation between chlorophylls and soluble sugars concentrations in leaves. For instance, while chlorophyll concentrations increased 15 dat in La-treated seedlings, sugar concentrations remained unchanged. Conversely, chlorophyll concentrations were unchanged or showed a decrease in response to La 30 dat, whereas total soluble sugars significantly increased (Table 2; Figure 7). Photosynthesis and source-to-sink transport of sugars are significantly regulated by environmental factors, including mineral deficiencies, and soil pollutants (Lemoine et al., 2013). Furthermore, source and sink can communicate interactively and exert mutual influences on each other (Bobeica et al., 2015). For instance, when the source-to-sink ratio is reduced, the leaves (source) can increase leaf efficiency toward a compensation of their photosynthetic rate to meet the demand of fruit development (sink; Petrie et al., 2003; Kliewer and Dokoozlian, 2005). Bobeica et al. (2015) observed that source limited plants increased their net CO_2_ exchange rate per unit of leaf area compared to source sufficient plants, which may also influence leaf chlorophyll contents. Moreover, the amount of sucrose available for export from source leaves depends on key factors such as CO_2_ fixation, partitioning between starch synthesis in the chloroplast and triose-phosphates exported from the chloroplast for sucrose synthesis, and transient storage of sucrose in the vacuole. If one of these factors is modified, the amount of sucrose available for export is affected and therefore source/sink relationships can be altered (Lemoine et al., 2013). Hence, the increase in sugar concentration in source leaves despite the reduced chlorophyll concentration observed 30 dat could result from a possible phloem transport decrease. Altogether, those results may consistently explain the responses we observed herein.

The higher foliar protein concentration in treatments with La^3+^ especially observed 15 dat in all four varieties evaluated (Figure 9) may be associated with an acceleration of the transformation of inorganic N to organic forms, such as proteins (Pang et al., 2002). Indeed, He et al. (2005) demonstrated that La^3+^ itself may enhance the mRNA and protein accumulation in lettuce, though the exact mechanisms of such enhancement triggered by La still remain unclear. The evident increase in protein concentration in La-treated seedlings 15 dat was negatively correlated with the concentration of amino acids during the same time of measurement in most varieties evaluated (Figure 8). Similarly, in soybean seedlings (10-day old), Huang et al. (2013) observed that La led to a 10.39% amino acid decrease, while the content of soluble proteins was increased by 12.93%, compared with those of the control. This response might be attributable to the feedback adjustment of reaction production (glutamine) in the glutamine synthetase (GS)/glutamate synthase (GOGAT) cycle (Mo et al., 2001). Nevertheless, 30 dat, La stimulated the amino acid concentration in three of the four varieties evaluated, while the concentration of proteins was increased in only two of the four varieties exposed to La, which may suggest a possible stress signal induced by La because of the time of exposure and the advanced age of the plants. Indeed, Kawano et al. (2002) found that La may trigger oxidative stress in a time- and dose-dependent manner in tobacco (*Nicotiana tabacum*) BY-2 cells, which is a typical effect of REE inducing hormetic dose-responses in plants. Accordingly, Wang et al. (2012b) reported that La at low concentrations (i.e., 2–8 μM LaCl_3_) promoted antioxidation against Cd stress in soybean seedlings, whereas at high concentrations (i.e., 30–480 μM LaCl_3_) it resulted in prooxidant effects, implying potential ecological risk. Moreover, Fashui et al. (2000, 2005) found that increasing the synthesis of ROS decreases the protein concentration. Since La may trigger both antioxidant and prooxidant effects depending on the time and concentration to which cells are exposed to it, this REE may modify plant metabolism, including amino acids and protein biosynthesis and turnover. Moreover, La influences N metabolism in soybean seedlings, since pretreatment with the La concentration considered optimal (20 mg L^−1^) increased the activity of the enzymes nitrate reductase (NR), GS, GOGAT, and glutamate dehydrogenase (GDH), and decreased the accumulation of nitrate and ammonium (Cao et al., 2007). This means that there was an efficient transformation of inorganic N into proteins promoted by the addition of La (Pang et al., 2002). Also, by adding La^3+^ (0.01, 0.1, 1, 10, and 100 mg L^−1^) to the culture medium to evaluate the development of purple coneflower (*Echinacea angustifolia*) calluses, a positive relationship between growth and soluble protein content was found, with the highest protein content being obtained with the La^3+^ dose of 0.1 mg L^−1^ (Ma et al., 2010).

Our results confirm that the application of a 10 μM La concentration in the nutrient solution, particularly during the first 15 d, increases the quality of sweet bell pepper seedlings. Seedling height, stem diameter, as well as concentrations of total soluble proteins and chlorophylls *a, b* and total were all increased in response to La in most if not all varieties evaluated, during our first measurements (i.e., 15 dat). On the other hand, means of plant height, stem diameter, and concentrations of total soluble sugars, soluble amino acids and proteins were in general improved in La-treated seedlings 30 dat. Since the initial growth stage in sweet bell pepper seedlings is the most critical phase for the commercial production of seedlings and fruits (De Grazia et al., 2004), La may play a pivotal role in horticulture. In terms of the stimulation of biomolecule synthesis in La-treated seedlings, it is worth noting that carbohydrate content determines the post-transplant growth rate, and stimulates plant height. Consequently, the enhanced plant height observed with the supply of La also results in increased sweet bell pepper seedling quality (Dufault, 1998). Importantly, La enhanced the number of bud flowers, which may have a great impact on pepper productivity during the reproductive stage employing La as a biostimulant. A biostimulant is by definition “a beneficial substance or compound other than primary, secondary, and micro plant nutrients that can be demonstrated by scientific research to be beneficial to one or more species of plants, when applied exogenously” (www.biostimulantcoalition.org; Colla and Rouphael, 2015). This means that La may trigger adaptive responses, including the boost of physiological, biochemical and molecular mechanisms that helps plants to overcome stresses or improve their performance under optimal conditions. It has been recognized that exposure of plants to a particular stress or stimulus may provide resistance to a stress of another kind or improve plant productive attributes (Puttonen, 1985, 1989), which may be the case in our study. In fact, La may change flavonoid synthesis in soybean seedlings under enhanced UV-B radiation by regulating the activities of phenylalanine ammonia-lyase (PAL), cinnamate-4-hydroxylase (C4H), 4-coumarate:coenzyme A ligase (4CL), and chalcone synthase (CHS), which is an enzymological mechanism governing the effect of La on flavonoid synthesis in plants under enhanced UV-B radiation (Fan et al., 2014).

Altogether, these beneficial effects may be explained, at least partially, by the fact that low doses of La can promote the cell membrane's transportation of nutrients (Zheng et al., 2002; Li et al., 2008). At the same time, plant cells may transport more nutrients that are used to maintain normal cell growth. Thus, as the intracellular nutrient elements' contents increase, both transcriptional and translational levels of molecules (i.e., genes and the corresponding encoded enzymes) involved in nutrient uptake and transport rise too (Xia et al., 2017), which is consistent with our experimental results in terms of total soluble proteins measured (Figure 8). We are currently investigating the impact of La on the nutrient status of these seedlings.

## Conclusions

The addition of 10 μM La in the nutrient solution increased plant height, stem diameter, and number of flower buds in most sweet pepper varieties evaluated. The beneficial effects of La were different, depending on the time of measurement (i.e., 15 or 30 dat) and varieties tested. Furthermore, the La treatment enhanced the concentration of biomolecules such as chlorophylls (15 dat), total soluble sugars (30 dat), total soluble amino acids (especially 30 dat), and total soluble proteins in leaves (30 dat) in most if not all varieties evaluated. Thus, La may improve quality parameters of sweet bell pepper seedlings. Since La may trigger hormesis, a dose-response phenomenon characterized by low-dose stimulation and high-dose inhibition, the possible impact of dosages and time of application on the whole metabolism of sweet bell pepper still remains at issue. In terms of horticulture, further research is required to find the right method of application (i.e., hydroponic solution, foliar spray, nanofertilizers, etc.), source, rate and phenological stage of La application for different pepper genotypes. In conclusion, La can provide outright stimulation to pepper and other crop species that might not occur with other beneficial elements. Hence, La is emerging as a potential biostimulator for agricultural proposes, since it can induce desirable plant responses in a hormetic manner. It is expected that the improvement in the quality of La-containing products and the advancement of the understanding of the biological mechanisms underlying the beneficial effects observed, would make the employment of La as biostimulant really beneficial in the near future.

Taking into consideration global trends and the challenges to sustainable development, special attention must be paid to an increasing population demanding more food and the negative impacts of climate change and deterioration of natural resources crucial for agriculture and food security. Deep understanding of the physiological, biochemical, and molecular mechanisms triggered by La as a beneficial element in this and other plants species under stress conditions may be a possible area of further study. Its potential role as a signaling mediator in response to environmental stimuli and stress factors could be of crucial significance for plants, providing the cell with spatial and transient information to perform better under challenging environmental conditions, or simply to improve plant production and productivity. The discovery and description of the molecular bases of such relationships would be a noteworthy contribution to the field of plant production. The aforementioned global challenges clearly justify further research on rare earth elements such as lanthanum.

## Author contributions

AGJ carried out the experiment and the measurements in laboratory. FCGM provided inputs for the study and edited the manuscript. OTS revised and edited the manuscript. LITT designed the study, did statistical analysis of the results, and prepared the first draft of manuscript and figures.

### Conflict of interest statement

The authors declare that the research was conducted in the absence of any commercial or financial relationships that could be construed as a potential conflict of interest.
