# Supplementary material for: Lanthanum Affects Bell Pepper Seedling Quality Depending on the Genotype and Time of Exposure by Differentially Modifying Plant Height, Stem Diameter and Concentrations of Chlorophylls, Sugars, Amino Acids, and Proteins
Source: Front Plant Sci. 2017 Mar 10;8:308. doi: 10.3389/fpls.2017.00308 (PMC5344905; doi:10.3389/fpls.2017.00308)
Supplement: Supplementary file 1 [file Table1.docx]

Supplementary Material

Lanthanum affects bell pepper seedling quality depending on the genotype and time of exposure by differentially modifying plant height, stem diameter and concentrations of chlorophylls, sugars, amino acids and proteins

Atonaltzin García-Jiménez, Fernando Carlos Gómez-Merino, Olga Tejeda-Sartorius and Libia Iris Trejo-Téllez^*^

***Correspondence:** Libia Iris Trejo-Téllez. Colegio de Postgraduados Campus Montecillo. Soil Science. Laboratory of Plant Nutrition. Montecillo, State of Mexico, Mexico. C. P. 56230. tlibia@colpos.mx

**Supplementary Material 1.** Main agronomic descriptors of sweet bell pepper varieties evaluated.

| **Variety** | **Fruit shape /lobes per fruit** | **Fruit color/maturation** | **Fruit size** | **Reference** |
| --- | --- | --- | --- | --- |
| Sven | Blocky/4 | Dark green/Golden yellow | Large | Rijk Zwaan, 2014a |
| Sympathy | Blocky/4 | Orange/glossy | Large/extra large | Rijk Zwaan, 2014b |
| Yolo Wonder | Blocky/3 to 4 | Dark green/red | 4 to 4.5 inches | Rijk Zwaan, 2006 |
| Zidenka | Blocky/4 | Red | Large/extra large | Rijk Zwaan, 2010 |

**References**

Rijk Zwaan. (2006). Rijk Zwaan Sweet pepper-product types. July 9th, 2016. shttp://files.tlhort.com/topicassets/attachments/ta_102_alternative_pepper_products_from_rijk_zwaan.pdf

Rijk Zwaan. (2010). International Catalogue. 2010-2011. July 9th, 2016. http://files.tlhort.com/topicassets/attachments/ta_243_export_catalogue_2009-2010.pdf

Rijk Zwaan. (2014a). Sven Rz F1. July 8th, 2016. www.rijkzwaanusa.com/wps/wcm/connect/RZ+USA/Rijk+Zwaan/Products_and_Services/Products/Crops/Sweet+Pepper?pcpage=3&frm=1&varname=SVEN%20RZ%20F1%20(35-220)&his=c293LHVuZGVmaW5lZCwwO2hhcnYsdW5kZWZpbmVkLDA7cGxhbnQsdW5kZWZpbmVkLDA7cmFkaW9zY2hlZCxoYXJ2LDA7

Rijk Zwaan. (2014b.) Symphaty Rz F1. July 8th, 2016. www.rijkzwaanusa.com/wps/wcm/connect/RZ+USA/Rijk+Zwaan/Products_and_Services/Products/Crops/Sweet+Pepper?pcpage=3&frm=1&varname=SYMPATHY%20RZ%20F1&his=c293LHVuZGVmaW5lZCwwO2hhcnYsdW5kZWZpbmVkLDA7cGxhbnQsdW5kZWZpbmVkLDA7cmFkaW9zY2hlZCxoYXJ2LDA7
