# Supplementary material for: Lanthanum Affects Bell Pepper Seedling Quality Depending on the Genotype and Time of Exposure by Differentially Modifying Plant Height, Stem Diameter and Concentrations of Chlorophylls, Sugars, Amino Acids, and Proteins
Source: Front Plant Sci. 2017 Mar 10;8:308. doi: 10.3389/fpls.2017.00308 (PMC5344905; doi:10.3389/fpls.2017.00308)
Supplement: Supplementary file 2 [file Table2.docx]

Supplementary Material

Lanthanum affects bell pepper seedling quality depending on the genotype and time of exposure by differentially modifying plant height, stem diameter and concentrations of chlorophylls, sugars, amino acids and proteins

Atonaltzin García-Jiménez^1^, Fernando Carlos Gómez-Merino^2^, Olga Tejeda-Sartorius^3^ and Libia Iris Trejo-Téllez^1,3^

***Correspondence:** Libia Iris Trejo-Téllez. Colegio de Postgraduados Campus Montecillo. Soil Science. Laboratory of Plant Nutrition. Montecillo, State of Mexico, Mexico. C. P. 56230. tlibia@colpos.mx

Supplementary Material 2. Summary of main effects triggered by lanthanum in horticultural crops.

| **Crop species** | **System tested** | **[La] evaluated** | **Source of La** | **Authors** | **Main effects observed** |
| --- | --- | --- | --- | --- | --- |
| *Spinacea oleracea* | Thylakoid suspensions | 2 mM (2000 µM) | LaCl_3_ | Chen and Tao, 2001 | La inhibits O_2_ evolution in the PSII, by substituting Ca^2+^, thus reducing oxidative stress. |
| *Spinacea oleracea* | Hydroponics | 10 mL L^-1^ (15.39µM) | La(NO_3_)_3_ | Hong et al., 2002 | La stimulates plant growth, increases chlorophyll contents and induces photosynthetic rate. |
| *Spinaces oleracea* | Hydroponics | 30-60 mg L^-1^ (92.33-184.66 µM) | La(NO_3_)_3_ | Yan et al., 2005 | La stimulates the Hill reaction, as well as the enzymatic activity of Mg^2+^ -ATPasa and Ca^2+^-ATPasa. |
| *Lactuca sativa* | Tissue culture (*in vitro*; MS medium) | 0.04 mg L^-1^ (0.123 µM) | La(NO_3_)_3_ | He et al., 2005 | La reduces Cd accumulation by inducing transcription and translation of phytochelatin genes. |
| *Cucumis sativus* | Hydroponics | 0.02-2.0 mM (20-2000 µM) | LaCl_3_ | Zeng et al., 2000 | La reduces concentrations of Na, Mg, Cl, K and Ca, but increases those of Mn and Fe. |
| *Cucumis sativus* | Hydroponics | 0.002-2 mM  (2-2000 µM) | - | Huang et al., 2003 | La changes the absorption of oligoelements. |
| *Cucumis sativus* | Hydroponics | 0.002-2 mM  (2-2000 µM) | LaCl_3_ | Shi et al., 2006 | La stimulates chloroplast formation |
| *Cucumis sativus* | Hydroponics | 0.002-0.02 mM (2-20 µM) | LaCl_3_ | Shi et al., 2005 | La regulates the expression of antioxidant enzymes (PO, CAT and SOD) during plant growth. |
| *Capsicum annuum*  *Solanum lycopersicum* | Soil fertilization | 0.001-0.0015 M (1000-1500 µM) | La_2_(SO_4_)_3_ | Abasheeva et al., 2003 | La increases fruit yield, as well as concentrations of proteins, sugars and ascorbic acid in fruits. |
| *Capsicum annuum* | Hydroponics | 1 mg L^-1^ (4.08 µM) | LaCl_3_ | Hu et al., 2014 | La ameliorates plant responses to salt stress, by regulating methylation. |
| *Solanum lycopersicum* | Hydroponics | 0.3 µM | La(NO_3_)_3_ | Wu et al., 2013 | La affects amino acid concentrations. |
| *Solanum lycopersicum* | Foliar spray | 10-20 mg L^-1^ (40.77-81.54 µM) | LaCl_3_ | Xie et al., 2014 | La increases biomass dry weight and reduces Cd concentrations in leaves, stems, roots and fruits. |

References

Abasheeva, N., Kozhevnikova, N. M., Merkusheva, M. G., Boloneva, L. N. and Soldatova, Z. (2003). Increasing the nitrification capacity of chestnut soil under the influence of lanthanum-containing microfertilizers. *Chem. Sust. Dev*. 11, 705-710.

Chen, W.-J., and Tao, Y. (2001). The EXAFS study on the local structure of lanthanum in spinach PSII. *Biol. Trace Elem. Res.* 82, 231-237. doi: [10.1385/BTER:82:1-3:231](https://dx.doi.org/10.1385/BTER:82:1-3:231)

He, Z., Li., Zhang, H., and Ma, M. (2005) Different effects of calcium and lanthanum on the expression of *Phytochelatin synthase* gene and cadmium absorption in *Lactuca sativa*. *Plant Sci.* 168, 309-318. [doi: 10.1016/j.plantsci.2004.07.001](http://dx.doi.org/10.1016/j.plantsci.2004.07.001)

Hong, F., Wei, Z., and Zhao, G. (2002). Mechanism of lanthanum effect on chlorophyll of spinach. *Sci. China C Life Sci.* 45, 166-176. doi: 10.1360/02yc9019

Hu, N., Sui, Y., Cai, Y., Fan, H., and Lin, Y. (2014). Effects of lanthanum on POD expression and DNA methylation of purple pepper under salt stress. *J. Rare Earth*. 32, 467-475. doi: 10.1016/S1002-0721(14)60095-8

Huang, Z., Chen, G., and Du, J. (2003). Influence of lanthanum on the uptake of trace elements in cucumber plant. *Biol. Trace Elem. Res.* 95, 185-192. doi: [10.1385/BTER:95:2:185](https://dx.doi.org/10.1385/BTER:95:2:185)

Shi., P., Chen, G. C., and Huang, Z. W. (2005). Effects of La^3+^ on the active oxygen-scavenging enzyme activities in cucumber seedling leaves. *Russ. J. Plant Physiol.* 52, 294–297. doi: 10.1007/s11183-005-0044-3

Shi, P., Huang, Z., and Chen, G. (2006). Influence of lanthanum on the accumulation of trace elements in chloroplasts of cucumber seedling leaves. *Biol. Trace Elem. Res.* 109, 181-187. doi: [10.1385/BTER:109:2:181](https://dx.doi.org/10.1385/BTER:109:2:181)

Wu, J., Chen, A., Peng, S., Wei, Z., and Liu, G. (2013). Identification and application of amino acids as chelators in phytoremediation of rare earth elements lanthanum and yttrium. *Plant Soil* 373, 329-338. doi: 10.1007/s11104-013-1811-0

Xie, W., Xiong, S., Xu, W., Chen, R., Zhang, J., and Xiong, Z. (2014). Effect of exogenous lanthanum on accumulation of cadmium and its chemical form in tomatoes. *Wuhan Univ. J. Nat. Sci.* 19, 221-228. doi: 10.1007/s11859-014-1005-5

Yan, W., Yang, L., and Wang, Q. (2005). Distribution of lanthanum among the chloroplast subcomponents of spinach and its biological effects on photosynthesis: location of the lanthanum binding sites in photosystem II. *Chin. Sci. Bull.* 50, 1714-1720. doi: 10.1360/982004-876

Zeng, F. L., Shi, P., Zhang, M. F., and Deng, R. W. (2000). Effect of lanthanum on ion absorption in cucumber seedling leaves. *Biol. Trace Elem. Res.* 78, 265-270. doi: [10.1385/BTER:78:1-3:265](https://dx.doi.org/10.1385/BTER:78:1-3:265)
